# How do emotionally intelligent individuals react to other people’s emotions? A study on emotional and facial reactions

**DOI:** 10.1186/s40359-025-02425-5

**Published:** 2025-03-10

**Authors:** Christelle Gillioz, Maroussia Nicolet-dit-Félix, Sylvain Delplanque, Marcello Mortillaro, David Sander, Marina Fiori

**Affiliations:** 1 Swiss Federal University for Vocational Education and Training, Lausanne, Switzerland; 2 Cognitive Science Center, University of Neuchâtel, Neuchâtel, Switzerland; 3 Swiss Center for Affective Sciences, University of Geneva, Geneva, Switzerland

**Keywords:** Emotional intelligence, Emotional contagion, Facial mimicry, Hypersensitivity, Emotion information processing

## Abstract

**Background:**

According to the hypersensitivity hypothesis, highly emotionally intelligent individuals perceive emotion information at a lower threshold, pay more attention to emotion information, and may be characterized by more intense emotional experiences. The goal of the present study was to investigate whether and how emotional intelligence (EI) is related to hypersensitivity operationalized as heightened emotional and facial reactions when observing others narrating positive and negative life experiences.

**Methods:**

Participants (144 women) watched positive and negative videos in three different conditions: with no specific instructions (spontaneous condition), with the instructions to put themselves in the character’s shoes (empathic condition) and with the instructions to distinguish themselves from the character (distancing condition). The activity of the corrugator supercilii and zygomaticus major muscles was recorded and after each video, the participants reported the arousal corresponding to their emotion during the video. The EI facets emotion recognition (ER), emotion understanding (EU), and emotion management (EM) were measured.

**Results:**

Participants’ self-reported arousal and facial motor responses increased in the empathic condition compared to the spontaneous condition and then decreased in the distancing condition. Although there was no effect of EI on reported arousal, EI, specifically EU and EM, seemed to influence facial reactions during the task. In the spontaneous and empathic conditions, EU was associated with a greater difference in zygomaticus activation between positive and negative videos, suggesting that individuals high on this EI facet may react more to positive emotion of others. In the spontaneous and distancing conditions, EM predicted less corrugator activation when watching negative videos, suggesting that individuals high on this EI facet may spontaneously regulate their negative emotions.

**Conclusions:**

This study suggests that hypersensitivity effects might better be captured by implicit measures such as facial reactions rather than explicit ones such as reporting of emotion. They also suggest that some EI facets and viewing conditions (spontaneous, empathic, and distancing view) influence emotional facial reactivity.

**Supplementary Information:**

The online version contains supplementary material available at 10.1186/s40359-025-02425-5.

## Introduction

The experience of affect in human beings encompasses a complex interplay of physiological, cognitive, and behavioral responses to emotional stimuli, both internal and external. Particularly significant is the phenomenon of affective responses elicited by the emotions of others, which plays a pivotal role in social interactions and interpersonal relationships. In this paper, we report a study investigating one factor that comprises a set of skills enabling individuals to navigate social interactions effectively and that may be implied in the propensity to react to others’ emotions, namely emotional intelligence (EI). In the next sections, we first present emotional contagion and emotional mimicry, then we introduce the construct of EI and its recent conceptualization, to finally present the rational of this study.

### The experience of affect in relation to the emotions of others

Empathy, emotional contagion, and emotional mimicry are related yet distinct fundamental concepts in understanding individual responses to emotions of others. Empathy is broadly defined as the capacity to imagine, experience, and understand what another person is feeling [1]; it involves both cognitive perspective-taking and affective resonance. Emotional contagion describes the spontaneous or deliberate sharing of emotions, including verbal and nonverbal emotional behavior, perceived in others [2]. More specifically, emotional contagion corresponds to an “affective state that matches the other’s emotional display” ( [3], p. 130). Facial emotional mimicry corresponds to a congruent facial response to another person’s emotional expression [4], which can be overt (e.g., when someone smiles in response to someone else smiling) or covert (i.e., corresponding to very subtle activations of muscles congruent with someone else’s expression). According to the embodied simulation theories [5, 6], through mimicking, one can simulate what the other is feeling by reactivating somatosensory, interoceptive and motor content implicated in emotion, leading to the understanding of the other’s emotion. In other terms, recognizing and understanding someone else’s emotion entails the use of one’s own bodily and neural states to access the emotion concepts. In this sense, the simulation of other’s emotion through corresponding facial muscles activation would be a precursor to emotional contagion. The relationship between emotional contagion and emotional facial mimicry is however still a much-debated topic in the literature, with studies investigating this link leading to inconsistent results [3, 7, 8, 9].

Hess and Blairy [3] for example showed that participants mimicked the facial expressions of emotional stimuli they had to evaluate, and that the participants’ emotional state was influenced by these facial expressions (i.e., emotional contagion). However, mediation analyses did not support the hypothesis that emotional contagion was predicted by mimicry. More recent studies have reported different results, which tend to support a (partial) relationship between mimicry and emotional contagion. Olszanowski et al. [10] for instance found (1) that being exposed to a specific emotion predicted congruent emotional mimicry in their participants, (2) that emotional mimicry predicted the participants’ emotion, and (3) that there was a direct link between the emotion presented and the participant’s emotion. Crucially, this last relation was reduced when controlling for emotional mimicry, suggesting that mimicry partially explains emotional contagion. In the same vein, Lischetzke et al. [11] reported increased unpleasant mood in participants exposed to sad stimuli, and, more importantly, showed that this increase in unpleasant mood was predicted by stronger mimicry, supporting the idea that mimicry and emotional contagion are related.

All in all, it appears that mimicry and emotional contagion share some common bases even though their relation might not be apparent in all studies, probably because of differences in methods and measures. Emotional contagion may therefore be conceptualized as a multilevel phenomenon characterized not only by emotional mimicry, but also by the full-fledged emotional experience of psychological feelings. In this view, mimicry (i.e., the activation of similar motor or autonomic responses in oneself in response to another person’s emotional expressions) may facilitate emotional contagion, which in turn may lead to empathy and the capacity to understand others’ feelings [12].

Importantly, emotional contagion and mimicry have been found to be moderated by individual differences.

#### Individual differences in emotional contagion and mimicry

Past research has demonstrated that individuals with high levels of narcissism are less prone to emotional contagion of positive emotions [13] or that individuals with higher levels of emotion recognition accuracy display stronger facial mimicry than those with lower capacities [14, 15]. Studies have also shown that empathy influenced mimicry, that is, individuals with higher empathy capacities responded with stronger congruent facial muscles responses when presented with facial expressions [16, 17, 18]. Additional moderators of emotional contagion include individual differences in people’s attention, perceptions of interdependence and dispositional susceptibility to emotional contagion [19].

Interestingly, several factors that have been shown to influence emotional contagion and mimicry are also involved in emotional intelligence (EI), which we present and discuss in the next section.

### Emotional intelligence

Different conceptualizations of EI are present in the scientific literature. In this paper, we focus on ability-EI, based on Salovey and Mayer’s seminal work, which considers EI as a form of intelligence related to different EI facets ( [20]; refined in [21]): the recognition, understanding and management of emotions. Ability-EI can be measured with maximum-performance tests, where individuals’ capacities regarding the different facets are evaluated. For instance, emotion recognition can be assessed in tests presenting emotional expressions that must be labelled such as the Geneva Emotional Recognition Test [22].

#### Dual-process accounts of EI

Recently, it has been proposed that assessing ability-EI based on maximum-performance tests might not fully account for how emotionally intelligent individuals perform in different circumstances [23]. Relying on the theoretical framework of dual-process models (e.g., [24]), which considers both conscious and automatic processes to explain behavior, it is argued that EI is not only based on conscious processes and thoughts such as the abilities to recognize typical facial expressions or the knowledge concerning the best reaction that one can have in an emotional situation, but also on more automatic, less conscious processes, such as attentional capture by emotional stimuli, fast recognition of subtle emotional cues (e.g., the detection of microexpressions), or actual physiological reactivity to an emotional situation [25, 26].

Following this idea, EI has been described as based on two different yet related components [23]. The first one, emotional knowledge (EI_K_) corresponds to conscious reasoning capacities about emotions. It is based on general knowledge about emotions and reflects top-down processing of emotion. The second one, emotion information processing (EI_P_), relates to more automatic bottom-up processing of emotional information. Current tests of ability-EI are more suited to capture the former, whereas cognitive tasks that tap into the processing of emotion information, such as attentional bias towards emotion information or emotional reactivity, may better assess the latter component. Another way to consider this distinction is by referring to the different components of intelligence [27]: EI_K_ can be viewed as the crystallized component of EI, reflecting what individuals know about emotions and EI_P_ as a fluid component, reflecting how individuals react to emotion or, in other terms, their sensitivity to emotion. Supporting this, previous research has shown that EI_K_ and EI_P_ are correlated with each other and can be conceptualized, within an overall EI factor, as separate components related respectively to crystallized and fluid intelligence [23].

#### EI and emotional hypersensitivity

Individual differences in EI_P_ can be envisioned on a continuum between low and high sensitivity to emotional information, with people having high level of EI_P_ being described as “hypersensitive” to emotion (see [26] for more information regarding EI_P_ and its relationship with hypersensitivity). Hence, hypersensitivity is introduced as a fundamental characteristic of EI, describing the way high EI individuals treat emotion information. More specifically, high-EI individuals, as compared to low, perceive emotion information at a lower threshold, pay more attention to emotion information, discriminate complex emotional expressions more easily and more quickly, and may be characterized by more intense emotional experiences.

Two studies have experimentally confirmed that EI_K_ is associated to hypersensitivity to emotion information. In a study using the dot-probe paradigm, high levels of EI_K_ predicted an attentional bias towards emotional stimuli: participants scoring high on the emotion understanding facet of EI_K_ (+ 1 standard deviation from the mean) were faster to respond to cues replacing emotional compared with neutral faces, which was not the case for individuals lower on this facet [28]. Another study provided evidence that high levels on the three facets of EI_K_ predicted better discrimination of complex emotional expressions in a task that demanded rapid and more instinctive emotion recognition in morphed faces [29]. The results of these two studies suggest that the hypersensitivity of high-EI individuals foster them to promptly perceive a higher quantity of emotion information that is generally more accurately discriminated.

This hypersensitivity phenomenon that up to date has been described at early stages of emotion information processing might modulate how individuals experience emotion, and how they react to the emotions experienced by others. As presented before, emotional contagion and mimicry are more important in individuals with higher levels of emotion recognition capacities, which is a component of EI. Emotional contagion also seems to share basic mechanisms with emotion understanding, another core component of EI. On this basis, it is fair to hypothesize that hypersensitivity related to EI might be involved in the subjective and physiological experience of affect in response to the emotions felt by another person, another step of emotion information processing. The goal of the current study was to evaluate whether, in a situation implying emotional contagion, EI_K_ may influence emotional reactivity measured through (1) ratings of arousal and (2) congruent facial muscles activation as an index of physiological experience. Specifically, we hypothesized that the experience of affect would be *more pronounced* in individuals who have higher emotion perception and emotion understanding capacities, because they are more prone to detect and correctly interpret subtle emotional stimuli in their environment. Importantly, EI is also characterized by an emotion management facet, which characterizes the knowledge people have on how to regulate their and other’s emotion. In association with this specific facet, we hypothesized that the experience of affect would be *less pronounced* for individuals with high emotion management abilities, specifically for negative emotional stimuli.

### The current study

In typical studies on emotional contagion, participants watch images or videos picturing individuals displaying specific emotional expressions and then report their affective state [3, 13]. Different emotional stimuli have been employed: static stimuli such as photographs of facial expressions of basic emotions [30] or dynamic stimuli, such as emotional expressions ranging in intensity or emotional scenes taken from movies [31]. Dynamic stimuli seem to induce more emotional response (e.g., [32]), probably because they are more ecological and have longer duration. Experimental studies have also varied the presentation time of the stimuli, for several seconds to several minutes (e.g., [13]) and longer time seem to promote contagion effect by reflecting everyday situations in which people are exposed to another person’s feeling. On this basis, in the present study, we presented our participants with stimuli that were especially tailored to promote emotional contagion.

We created videos displaying narrators sharing demographic characteristics with our participants (similar age, gender, occupation) who explained real emotional events—pleasant and unpleasant—that happened in their past. This allowed us to offer dynamic and social stimuli with situated context that aimed at increasing affiliation between the participants and the narrators. Videos also contain multimodal cues of emotions (i.e., facial expressions, vocal inflections, body language) and last for a longer time than pictures, which should render emotional contagion more likely. Moreover, we decided to use mildly intense emotional stimuli to maximize hypersensitivity effects. We thought that highly intense emotional stimuli might lead to emotional contagion in a general manner, independently of EI, whereas mildly emotionally intense stimuli might be more likely to elicit emotional contagion only in individuals with high levels of EI.

Following the videos, participants rated the valence of their emotion felt while watching and their level of arousal. This latter rating was retained as a subjective measure of emotional contagion.

In addition to emotional contagion, we also measured the activation of specific facial muscles over time with facial electromyography (fEMG; e.g., [3]). Research using fEMG has mainly investigated reactions to happiness, anger and sadness and has shown that participants reacted with increased activity in zygomaticus major (i.e., smiling muscle) in response to happiness and increased activity in corrugator supercilii (i.e., frowning muscle) in response to anger and sadness [4, 33]. Importantly, evidence is lacking to allow identifying specific patterns of facial muscles activation associated with discrete specific emotions and it is more appropriate to speak of valence-congruent facial responses such as increased activity in zygomaticus major for positive emotions and increased activity in corrugator supercilii for negative emotions [4, 11, 33]. Accordingly, we employed electrodes placed on the zygomaticus major to detect reactions to pleasant emotions and on the corrugator supercilii to detect reactions to unpleasant emotions. Reactions of these facial muscles were considered as an indicator of the level of spontaneous emotional reactivity in response to others’ emotions.

We manipulated the instructions given to the participants in order to disentangle what individuals tend to do naturally and what they are capable of, depending on their level on the different EI_K_ facets. Hence, in addition to a spontaneous viewing condition (spontaneous condition), we created two different conditions, one aiming at enhancing emotional contagion by asking the participants to put themselves into the character’s shoes (empathic condition), and the other one aiming at regulating emotional contagion by asking the participants to distinguish themselves from the character (distancing condition). The empathic condition was specifically intended to observe effects of emotion understanding and emotion perception, while the distancing condition was specifically intended to observe effects of emotion management.

#### Study hypotheses

The hypotheses of this study are aligned with the idea that EI functions through a form of emotional hypersensitivity, which in the current case corresponds to stronger reactions in terms of subjective emotional reactivity and congruent facial muscles activation, particularly for the EI_K_ facets of emotion understanding and emotion perception. Regarding these facets, we expected that, in the spontaneous condition, if individuals high on emotion understanding and emotion perception feel emotion more intensely, then these individuals should report more arousal than low-EI individuals for both positive and negative videos. This would mean that, spontaneously, people high on these facets react more intensely to emotions of others without instructions (H1). Another way to test this idea was to compare the reported arousal to both positive and negative videos between the spontaneous condition and the empathic one, with the hypothesis that individuals high on emotion understanding and emotion perception should show less differences between these conditions than individuals low on these facets (H2). Again, this would show that high EI individuals tend to naturally have more emotional contagion than low EI ones.

Regarding the EI_K_ facet of emotion management, we expected individuals high on this facet to have more capacities to regulate emotions, in an automatic or more conscious manner. For this reason, we hypothesized that more emotion management leads to more capacity to regulate emotion when needed. Importantly, we expected different patterns related to positive and negative videos: emotion regulation may not be necessarily activated when watching positive videos, contrary to when watching negative videos. Consequently, in the spontaneous condition, we expected an interaction between emotion management and valence, showing that people high on emotion management report less arousal for negative compared to positive videos and that this difference is larger than for people low on emotion management (H3). Comparing the spontaneous and distancing conditions, for negative videos, we hypothesized that individuals high on emotion management would have less difference in arousal between conditions than individuals low on emotion management (H4). For positive videos however, individuals high on emotion management should have more difference in arousal between the spontaneous and distancing conditions than individuals low on emotion management (H5).

We had the same hypotheses for ratings of arousal related to the emotions felt when watching the videos, and for spontaneous facial muscles activation congruent to the other person’s emotions measured through facial EMG.

## Method

### Participants and general procedure

We were not able to conduct a power analysis for this study, because we did not have the necessary information regarding estimates of effect sizes or how our independent variables would be distributed. Therefore, and as indicated in the study’s pre-registration, we aimed to test at least 130 participants in order to obtain normal distributions for the independent variables of the study (EI_K_ facets).

One hundred and forty-six women (mostly students), aged between 18 and 36 years (*M* = 23.16) and recruited at a Swiss University took part in this study. We decided to test only women to maximize their chance to empathize with the narrators in the video (also women) and to allow us to reduce the number of variables to control when investigating individual differences. The participants first had to complete an online testing session in which they filled in several questionnaires and tests and provided demographic information. In the week following the first session, the participants came to the lab where they had to complete the emotional contagion task. The study was approved by the Ethical Committee of the University of Geneva and all participants gave their informed consent. As the whole study lasted for approximately two hours, the participants were remunerated 45 CHF (approximately 50 USD). Due to technical problems, EMG data for two participants could not be collected, resulting in final sample sizes of 146 participants for the subjective data and 144 participants for EMG data.

### Questionnaires

The following measures were collected in the online session, in addition to other measures not presented in the present paper (listed in the Supplemental material).

#### The situational test of emotion understanding-brief

The situational test of emotion understanding-Brief (STEU-B [34]), is a performance-based test, comprising 19 items evaluating the understanding of emotion, through the presentation of short scenarios. Respondents read the short scenarios and then decide which of five emotions best matches the emotion felt by the protagonist. For example, for the item “John completes a difficult task on time and under budget. John is most likely to feel?”, the correct answer is “Pride”. There is one correct response to each item. Participants thus obtain a score of 1 for correct response, and 0 for incorrect response. The test-retest reliability for the full version of the STEU is 0.72 [35].

#### The situational test of emotional management-brief

The situational test of emotional management-Brief (STEM-B [36]), is a performance-based test that comprises 18 items evaluating how well the respondent knows how to manage emotions in different kinds of situations. Respondents are presented with short scenarios and have to select the most appropriate way to handle the issues presented and/or to manage the protagonist’s emotion. An example of item is “Juno is fairly sure his company is going down and his job is under threat. It is a large company and nothing official has been said. What action would be the most effective for Juno?”, for which the most effective choice is “Find out what is happening and discuss his concerns with his family.” For each item, the score corresponds to a weight derived from expert ratings. The test-retest reliability for the full version of the STEM is 0.85 [35].

#### The Geneva emotion recognition test short version

The Geneva emotion recognition test short version [37] contains 42 video clips with sound in which professional actors portray 14 different emotions. For each clip, respondents select which of the 14 emotions was portrayed. There is one correct answer to each clip. In our sample, Cronbach’s alpha was 0.68 and McDonald’s omega was 0.71.

### Emotional contagion task

#### Emotional video clips

The stimuli used in this experiment were created by the two first authors to obtain videos fulfilling the four following requirements: (1) French-speaking (2) female narrators between 18 and 35 years old, who talk about (3) moderately intense (4) positive and negative experiences. The complete procedure used to create the videos can be found in the supplementary materials. In a nutshell, we recorded 36 videos of women sharing positive and negative emotional experiences. A subset of 12 videos was then selected by the authors on the basis of their duration and their content. The pre-selected videos were then evaluated regarding their intensity and their valence by 40 French-speaking women aged between 19 and 34 (20 evaluators for the negative videos and 20 for the positive videos) on the online platform Prolific. Based on these evaluations, 6 videos (3 positives and 3 negatives) were selected to be included in the emotional contagion experiment. The selected positive and negative videos had similar ratings of valence, (*M*_*pos*_ = 89.63, *sd*_*pos*_ = 13.1, *M*_*neg*_ = 76.33, *sd*_*neg*_ = 20.9, *t*(2.12) = -2.92, ns), intensity (*M*_*pos*_ = 80.4, *sd*_*pos*_ = 17.3, *M*_*neg*_ = 73.4, *sd*_*neg*_ = 22.1, *t*(3.97) = -1.99, ns.) and touching potential (*M*_*pos*_ = 69.8, *sd*_*pos*_ = 22.5, *M*_*neg*_ = 61.0, *sd*_*neg*_ = 27.6, *t*(3.92) = -2.47, ns.). Their duration was between 87 and 125 s. More information on the videos can be found in Supplementary Table 1.

An additional neutral video was recorded. The female narrator read, in a neutral way, a journalistic text about cloud classification and how meteorologists observe the sky and elaborate weather forecast. This video had a duration of 124 s and was used as a first stimulus for all participants. This video was thought to be a baseline video to control for muscle activation in a neutral condition. However, half of our participants had greater muscle activation during this video than during the experimental ones. Therefore, we decided not to use it as a baseline. Moreover, since we are comparing muscle activation in different conditions for the same individuals, subtracting the baseline would not change the results. Eventually, this video was more a practice trial, helping our participants to understand the task.

#### Procedure and measures

The participants were tested individually in a quiet room. They were explained the task, i.e., that they would watch videos and follow specific watching instructions for each video. The instructions started with “Please watch the video…” and depending on the condition followed by “in a natural manner” (spontaneous condition), “while adopting an internal perspective, by putting yourself in the narrator’s shoes” (empathic condition), or “while adopting an external perspective, by trying to distinguish yourself from the narrator” (distancing condition).

The experiment started with the neutral video with the instructions from the spontaneous condition. Then the experimental videos were presented, starting either with the positive or the negative ones and in the following fixed condition order: spontaneous, empathic, and distancing. Each participant watched six videos in total, one video in each condition. Among the sets of positive and negative videos, the videos were presented in counterbalanced order across the participants to avoid a confound between the video itself and the watching condition.

After each video, the participants had to evaluate the extent to which they managed to follow the instructions on a 5-point Likert scale (not at all, a little, moderately, well, completely). Then, they had to evaluate two aspects of their emotional state. First, their general state on a 100-point analog scale ranging from *negative (0.0)* to *positive (10.0)* with a middle point at *neutral*. Second, their arousal state on a 100-point analog scale ranging from *calm (0.0)* to *activated/agitated (10.0)* with a middle point at *neutral*. In a second step, they had to evaluate the same aspects of the narrator’s emotional state. We retained the evaluation of the participant’s arousal as a measure of emotional reactivity. Although the evaluations of the narrators’ emotional state by the participants might be of interest, it is not directly related to the research question we are investigating in this paper, i.e., the extent to which EI influences emotional reactivity.

### EMG data acquisition

#### Apparatus

Experimental events were controlled by PsychoPy Builder Version 2022.2.2 (Open Science Tools Ltd) and implemented on a computer with a Microsoft Windows operating system. The videos were displayed on a 17-inch computer screen from a viewing distance of about 70 cm. The videos were presented at 1088 pixels in height x 1920 pixel in width.

#### EMG recording

EMG recordings were taken for the corrugator supercilii and zygomatic major muscles on the left side of the face using disposable solid gel Ag/AgCl electrodes (Neurospec AG). Electrodes were positioned according to the guidelines of Fridlund and Cacioppo [38]. Before the placement of the electrodes, the skin was cleaned with alcohol. EMG signals were relayed through shielded cable to Biopac amplifiers (Biopac Systems, Inc., Santa Barbara, CA), where signals were amplified 5,000 x and digitized at 1000 Hz.

#### EMG preprocessing

EMG data were analyzed using AcqKnowledge^®^ 4.4.1 Software (Biopac System, Inc). Raw EMG data were preprocessed with a 20 to 400 Hz band-pass digital filter, rectified, low-passed filtered below 40 Hz and down sampled to 100 Hz. In order to exclude trials with excessive facial muscle activity, signals with a maximum amplitude higher than 1mV were visually inspected. When the amplitude was abnormally high on an extended duration, the trial was removed from further analyses.

### Statistical analysis

Data analyses were performed using R Version 4.3.0 [39] and followed a multiple step procedure. First, the data were cleaned prior to conducting analyses based on our pre-registration. As stated in the pre-registration, data from participants with scores on EI_K_ measures (STEU, STEM, GERT) falling below the median less 2.5 times the median absolute deviation (such as recommended in [40]) were excluded from the corresponding analyses. As the participants viewed videos in all conditions (within-subject design), we relied upon linear mixed effects models fitted with the lme4 [41] and lmerTest [42] R packages. As we had repeated measures, we included random intercepts for participants, but we did not include random slopes as there was only one measure per participant in each condition. All continuous independent variables were standardized around the grand mean. When all three conditions were included in the model, condition was treatment coded, with the reference set to the spontaneous condition. In all other cases, factors were sum-coded (-0.5, + 0.5), with the reference set to the spontaneous condition. We tested the influence of each facet of EI_K_ in separate analyses. Tables summarizing the models are available in the supplementary materials.

### Transparency and openness

We report how we determined our sample size, all data exclusions, all manipulations, and all measures in the study. This study’s design was preregistered, as were the hypotheses and analyses regarding the subjective measures of emotional contagion (see https://osf.io/q8t92). The pre-registration occurred during the data collection but before having looked at the data. The hypotheses regarding the fEMG measures were not pre-registered but are in the same vein as those related to the subjective ones. When we pre-registered hypotheses we were still uncertain with respect to how analyze the fEMG data and given the variety of available options we preferred not to commit to employ specific indexes of mimicry at that moment. All data used in this paper and analysis codes are available at https://osf.io/eb8qm.

## Results

### Evaluation of the videos’ ratings in the spontaneous condition

Before running the analyses, we checked that our participants shared the same judgments as the women who evaluated the films in the pre-test. In the spontaneous condition, the films should not differ regarding their arousal, which was confirmed, *F*(5, 282) = 1.54, *p* =.18. Relatedly, neither the positive videos (*F*(2, 141) = 0.79, *p* =.45) nor the negative videos (*F*(2, 141) = 0.18, *p* =.83 did differ regarding the valence evaluation.

### Participants’ success in following instructions

In general, our participants reported succeeding in following the watching instructions (*M* = 3.90, *SD* = 1.02) but not all instructions were equally followed as shown by a Friedman test, χ^2^(2) = 50.08, *p* <.001. Specifically, participants reported being less able to distinguish themselves from the character (*M* = 3.41, *SD* = 1.17) than putting themselves in their shoes (*M* = 4.08, *SD* = 0.87) or watching the video in a natural manner (*M* = 4.11, *SD* = 0.90). Importantly, none of the EI_K_ facets influenced these results.

### Self-reported emotional arousal during the videos

Before testing our hypotheses related to EI, we verified that the participants’ subjective arousal was influenced by the conditions of the experiment (see Table 1 for the reported arousal in the different conditions). We expected the subjective arousal to fluctuate depending on the watching instructions: it should increase in the empathic condition and decrease in the distancing condition compared to the spontaneous one. As expected, the participants’ reported arousal increased in the empathic (*β* = 0.49, 95%CI [0.17–0.81], *p* =.003) compared to the spontaneous condition. However, the decrease in the distancing (*β* = -0.16, 95%CI [-0.48–0.17], *p* =.34) compared to the spontaneous condition was not significant (see Supplementary Table 2). Of note, the reported arousal in the distancing condition was lower than in the empathic one (*β* = -0.65, 95%CI [-0.97– -0.32], *p* <.001, see Supplementary Table 3). These results show that emotional contagion was enhanced in the empathic condition compared to the other two conditions. They are also aligned with the participants’ report of their greater difficulty in following the instructions of the distancing condition.

**Table 1:** Means and standard deviations of the subjective reports of arousal in the three experimental conditions

Condition	Mean	SD
Spontaneous	4.59	2.33
Empathic	5.11	2.37
Distancing	4.45	2.48

We then tested the hypotheses related to the influence of EI_K_ on the participants’ reported arousal. We first examined the hypotheses implicating emotion understanding and emotion recognition. H1 predicted that in the spontaneous condition, arousal should increase with emotion understanding and emotion recognition. This was not the case, either for emotion understanding (*β* = -0.08, 95%CI [-0.37–0.21], *p* =.58, see Supplementary Table 4) or emotion recognition (*β* = 0.07, 95%CI [-0.22–0.36], *p* =.65, see Supplementary Table 5).

H2 derived from H1 and predicted that the increase in subjective arousal between the spontaneous and empathic condition should be larger for low-EI (emotion understanding and emotion perception) than for high-EI individuals. Neither the emotion understanding by condition interaction (*β* = 0.01, 95%CI [-0.31–0.34], *p* =.93, see Supplementary Table 6) nor the emotion recognition by condition interaction (*β* = -0.11, 95%CI [-0.44–0.22], *p* =.50, see Supplementary Table 7) were significant, meaning that the increase in subjective arousal between conditions did not depend on the level of these two facets.

The following hypotheses examined the role of emotion management in neutral and distancing conditions, considering the valence of the videos. H3 evaluated whether individuals higher on emotion management are naturally better at regulating emotions and therefore report less arousal for negative compared to positive videos in the spontaneous condition, with the difference in arousal between positive and negative videos being larger for these individuals than for those low on emotion management. The model did not show a significant interaction effect between emotion management and valence (*β* = 0.08, 95%CI [-0.43–0.59], *p* =.75, see Supplementary Table 8) on the reported arousal. In H4, we hypothesized that for negative videos, participants high on emotion management should show less difference in reported arousal between the spontaneous and distancing conditions than participants low on this facet; this would reflect the fact that they naturally downregulated their negative feelings without being asked to do so. However, the interaction effect between emotion management and condition (*β* = 0.15, 95%CI [-0.28–0.57], *p* =.50, see Supplementary Table 9) was not significant, which did not support the hypothesis. Finally, in H5 we hypothesized that for positive videos, participants high on emotion management should show more difference in reported arousal between the spontaneous and distancing conditions than participants low on this facet. Again, the model did not show a significant interaction between emotion management and condition (*β* = -0.09, 95%CI [-0.49–0.31], *p* =.67, see Supplementary Table 10).

### Facial muscles activation

EMG data were analyzed according to the same hypotheses as those used for the reported arousal. First, we tested whether the experimental manipulation influenced the participants’ facial muscles activation. We ran similar analyses to those presented earlier. We analyzed zygomaticus major’s and corrugator supercilii’s activation separately because we were not interested in comparing their activation, and we included the valence of the video in the model to consider the difference in muscle activation between positive and negative videos. It was indeed expected to see more zygomaticus activation in positive vs. negative videos and more corrugator activation in negative vs. positive videos. In a second step, we added EI_K_ in the models to investigate the influence of EI_K_ facets on facial muscles activation during the task. As for the analyses on subjective arousal, we investigated the influence of emotion understanding and emotion recognition in the spontaneous and empathic conditions and the influence of emotion management in the spontaneous and distancing conditions.

#### Zygomaticus major EMG

Given that the videos lasted between 87 and 125 s, we considered the mean activation of zygomaticus major during the video as the dependent variable^1^. According to the Box Cox test’s result [43], data were inverse transformed (-1/DV) to account for their distribution.

**Fig. 1:**
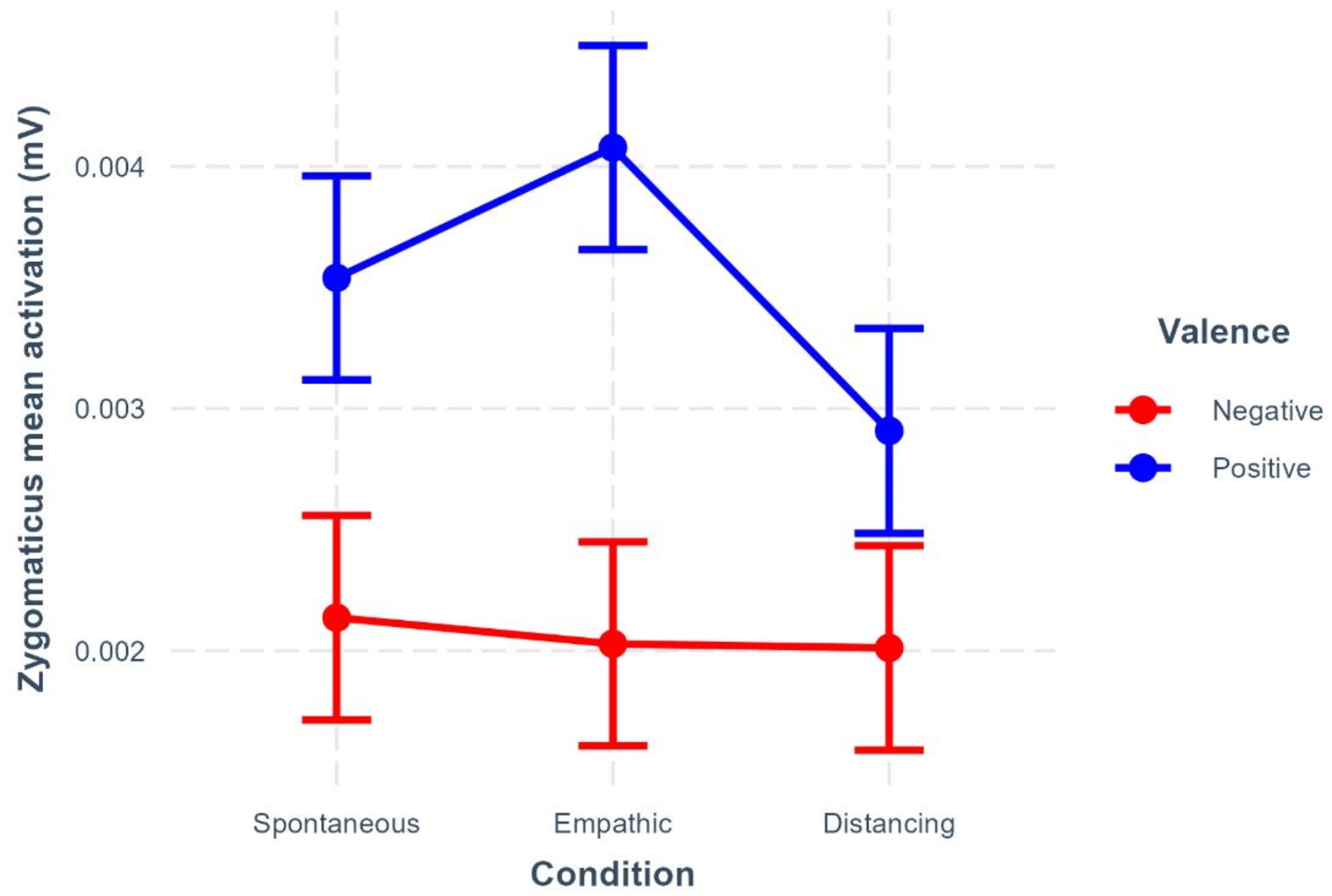
Zygomaticus mean activation in the different watching conditions depending on the valence of the video. Error bars represent 95% CIs

In a first model, we tested the effects of condition, valence, and their interaction, on zygomaticus activation. The model revealed a significant effect of valence (*β* = 178.38, 95%CI [158.98–197.78], *p* <.001), with more zygomaticus activation during the positive compared to the negative videos. More importantly, there was a significant interaction between condition and valence (see Fig. 1 and Supplementary Tables 11 and 12), showing that the difference in zygomaticus activation between positive and negative videos was larger in the empathic compared to the spontaneous condition (*β* = 64.05, 95%CI [16.61–111.50], *p* =.008) and smaller in the distancing compared to the empathic condition (*β* = -101.24, 95%CI [-148.78– -53.70], *p* <.001). There was however no significant difference between the distancing and the spontaneous condition (*β* = -37.19, 95%CI [-84.78–10.40], *p* =.13). This pattern is congruent with the results found in the analysis of subjective measures of emotional contagion and shows that the zygomaticus activation was larger in the empathic condition, when participants were asked to take the narrator’s perspective, compared to the other two conditions.

**Fig. 2:**
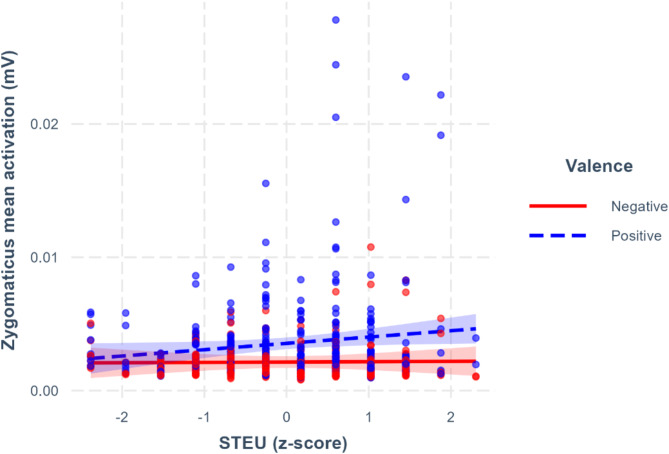
Zygomaticus mean activation for negative and positive videos as a function of emotion understanding. The intervals correspond to 95% CIs. The interaction is not statistically significant (*p* =.053)

In the following models, we tested the interaction of the different EI_K_ facets with valence and condition (spontaneous and empathic). The results revealed a tendency toward an interaction effect between emotion understanding and valence (*β* = 23.22, 95%CI [-0.28–46.72], *p* =.053, see Supplementary Table 13) but no three-way interaction. With increasing emotion understanding score, the difference in zygomaticus activation between positive and negative videos got larger, suggesting that, generally, participants with high levels of emotion understanding react with more facial muscles activation to positive videos than those low on this EI_K_ facet (Fig. 2).

When testing the influence of the other EI_K_ facets (emotion management or emotion recognition) no effect related to these variables appeared.

#### Corrugator EMG

Similarly to the analyses on zygomaticus, the dependent variable corresponded to the mean activation of the corrugator during the video. According to the Box-Cox test’s result [43], data were log-transformed to account for their distribution.

**Fig. 3:**
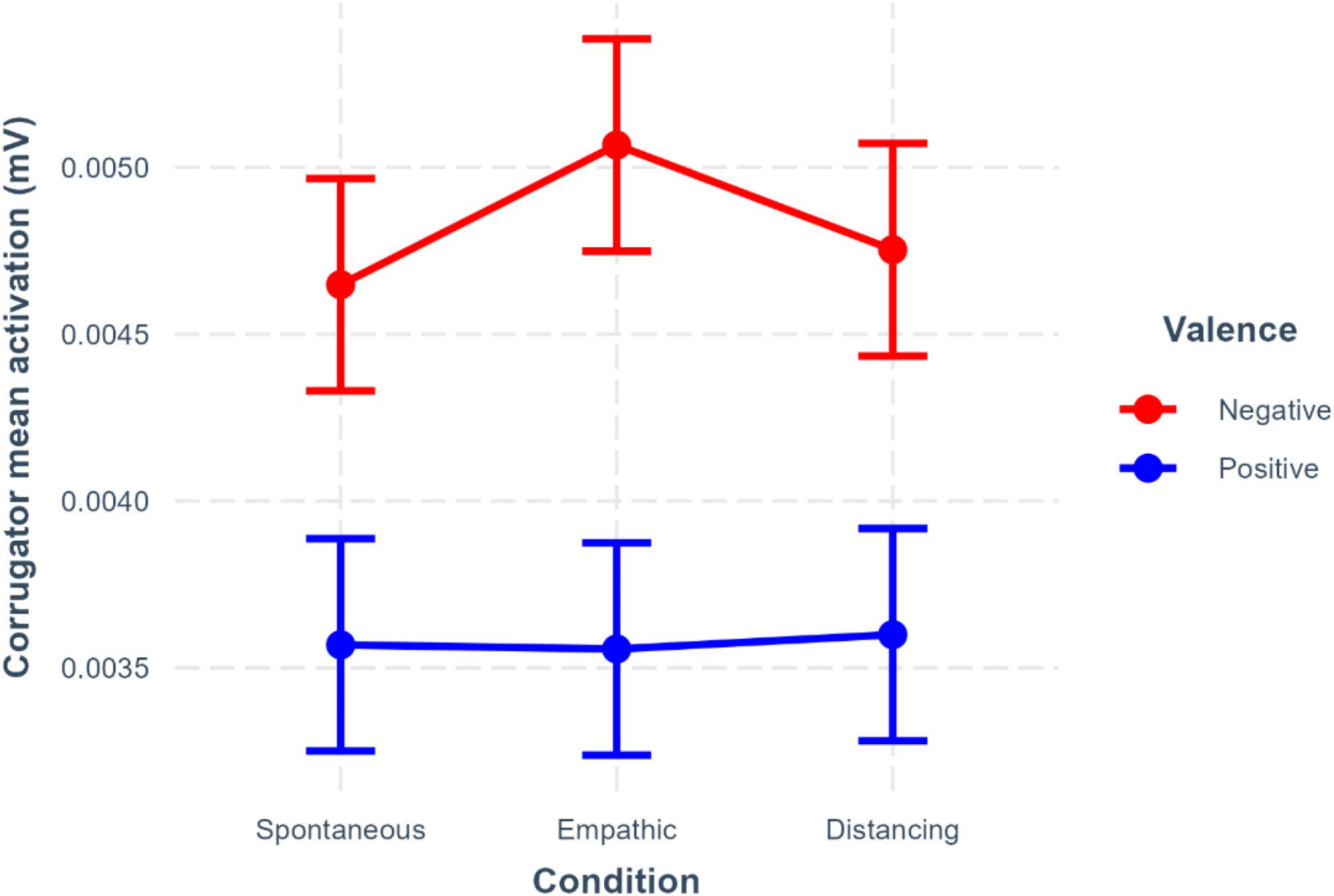
Corrugator mean activation in the different watching conditions depending on the valence of the video. Error bars represent 95% CIs. The interaction between valence and condition is not statistically significant

The first model testing the effects of valence, condition and their interaction on corrugator activation showed the expected effect of valence (*β* = -0.28, 95%CI [-0.34– -0.22], *p* <.001), with more activation during the negative than the positive videos (see Fig. 3). There was, however, no significant difference in corrugator activation between positive and negative videos in the empathic compared to the spontaneous condition (*β* = -0.06, 95%CI [-0.14–0.02], *p* =.17) or in the distancing compared to the spontaneous condition (*β* = 0.02, 95%CI [-0.06–0.10], *p* =.65, see Supplementary Table 14). Even though the visual inspection of the data tended to show the expected pattern, the difference in corrugator activation between the distancing and the empathic conditions only approached significance (*β* = 0.08, 95%CI [-0.01–0.16], *p* =.07, see Supplementary Table 15).

**Fig. 4:**
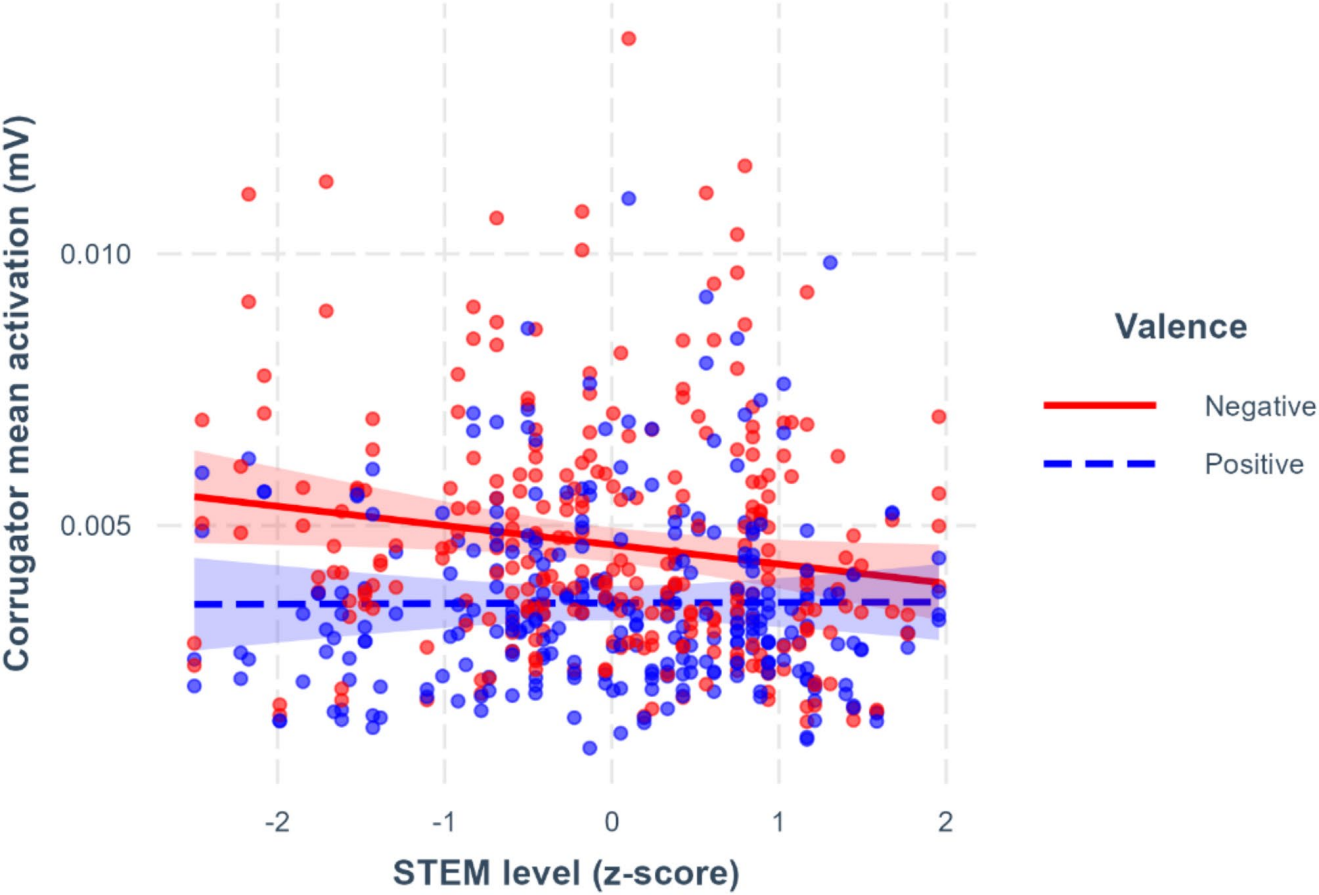
Corrugator mean activation for negative and positive videos as a function of emotion management. The intervals correspond to 95% CIs

In the following models, we tested the interaction of the different EI_K_ facets with valence and condition (spontaneous and distancing). The model including emotion management showed an interaction effect between this facet and valence (*β* = 0.07, 95%CI [0.02–0.11], *p* =.002, see Supplementary Table 16) but no three-way interaction. With increasing emotion management score, the difference in corrugator activation between positive and negative videos got smaller, suggesting that, generally, participants with higher levels of emotion management show less corrugator activation when watching negative videos than those lower on this EI_K_ facet (Fig. 4).

## Discussion

In this study, we tested whether the relationship between EI_K_ and EI_P_ at early stages of emotion information processing [28, 29] might also be present when observing reactions to emotions experienced by others. We designed a task in which participants watched videos presenting people narrating positive and negative personal emotional events. We manipulated the watching instructions of the videos in order to increase (empathic condition) or reduce (distancing condition) the participant’s emotional involvement, hence emotional contagion. We collected subjective measures of emotional reactivity (i.e., self-reported arousal) after watching the video and recorded activity of corrugator supercilii and zygomaticus major as indicators of more automatic reactions to others’ emotions when watching the video.

As expected, our manipulation did influence self-reported arousal, which increased from the spontaneous to the empathic condition and then decreased in the distancing condition. In contrast, the results regarding facial responses were not as clear. The expected valence effects were observed on both muscles, with more zygomaticus activation for positive relative to negative videos and more corrugator activation for negative compared to positive videos. Moreover, the zygomaticus showed the expected effects of our manipulation, with an increase in activation for positive videos between the spontaneous and the empathic conditions and then a decrease in the distancing condition. However, the corrugator activation was not influenced by the experimental conditions, suggesting that it was easier for the participants to regulate their reactions to others’ emotions when watching positive videos than negative ones.

Regarding the influence of EI_K_ on emotional contagion, results differed depending on whether it was measured through ratings of arousal or congruent facial muscles activation. We hypothesized that more emotional reactivity would be found for individuals with higher abilities on emotion understanding or emotion perception. With respect to self-reported emotional contagion, the data did not reveal any effect of these EI facets on the reported arousal during the task, either in the spontaneous viewing condition or in the empathic condition. With respect to facial muscles activation, the results suggested some influence of the emotion understanding facet on more automatic reactions to emotion, with a tendency to more zygomaticus activation associated to positive videos for participants high on this facet. A second hypothesis pertained to the effect of emotion management, for which different effects were expected, i.e., less emotional reactivity for negative videos in the spontaneous and distancing conditions for individuals high on this facet. This hypothesis was not supported by subjective reports of emotional contagion, but it was supported by more automatic affective responses: we found less corrugator activation during negative videos in participants high on the emotion management facet than in those low on this facet.

In all, the results obtained in this study are not supportive of the hypothesis that high EI leads to heightened emotional reactivity when subjective reports of emotional arousal are considered. However, when considering more automatic responses to others’ emotions, i.e., the activation of facial muscles implied in pleasant/unpleasant feelings, it appears that EI might modulate individuals’ reactions to emotion. Different explanations can be advanced at this point, regarding the discrepancy between the results on subjective arousal and on facial responses.

On a methodological point of view, in this study, we chose to employ videos that lasted for more than one minute, presenting relatable experiences from people resembling our participants, which, we thought, were well suited to favorize empathy processes in our participants. The moderate emotional intensity of the videos might nonetheless not have permitted to reveal “hypersensitivity” effects. When we chose to present mildly intense materials, we thought that they were more suited to show the role played by EI on emotional reactivity, as opposed to more intense stimuli that could have provoked strong reactions in all individuals. More recent theoretical considerations [44] have questioned this idea by advancing that strong reactions to *all* emotional stimuli, regardless of their intensity, cannot lead to effective adaptation. *Meaningful*, rather than undifferentiated, hypersensitivity to emotional stimuli, whereby one quickly identifies and reacts to emotional stimuli that are either intense or unexpected, might be more suited to explain how EI is associated to adaptive outcomes. Following this idea, being affected by mildly intense emotional experiences shared by strangers on video might indeed not reflect highly emotionally intelligent reactions. It should be noted that in another study in which we employed static emotional pictures of intense emotional expressions we were able to find that high EI individuals experienced and evaluated more extremely the intensity and arousal of angry and happy expressions. In particular, individuals with greater emotion understanding and perception evaluated angry expressions more extremely, while those higher on emotion management emphasized happiness more. Results were more consistent for intensity than arousal [45].

Another explanation, and the one that we favor, pertains to the relative lack of association between emotional contagion and facial emotional reactivity already described in the literature and that could be explained by how these concepts are measured, i.e., self-reports for the former and more objective measures for the latter. Here, we follow Olderbak and Wilhelm’s [46] argument that facial mimicry represents typical behavior that can be considered associated to an implicit empathic response to emotion perception. In contrast, emotional contagion measured with self-reports is based on more conscious processes, whereby one must think about their feelings in a retrospective way. Accessing and being able to report feelings and the associated arousal is not straightforward, notably because one has (1) to become conscious of their emotion and (2) to be able to verbalize it. As such, the feeling part of the emotional reaction does most probably not have a direct correspondence with the emotion underlying it and some aspects of this emotion might be lost when reporting it [47, 48]. In this case, small changes in the emotional experience, such as the ones that can be explained by individual differences in EI, might not be conscious enough to be reflected in self-reports of arousal.

On a related note, one aspect of EI is the capacity to regulate emotion. Following from this, it has been suggested that highly emotionally intelligent individuals are those who have high levels of EI_P_ (i.e., hypersensitivity) and can regulate this acute sensitivity [26]. It is then possible that, in our study, highly emotionally intelligent participants were in fact more prone to automatic emotional reactions, as suggested by their facial muscles activation, but were also more likely to automatically regulate their hypersensitivity, leading to no effect on the subjective ratings of their feelings. In other words, it could be that participants’ emotional reactivity was higher, but their subjective experience was not more intense because they were able to manage it quite unconsciously. In this case, subjective reporting of feelings might not allow to disentangle emotional reactivity and implicit emotion regulation, at least in the way we collected the measures in this study.

Interestingly, the emotion recognition facet of EI_K_ was not implicated in any effect in this experiment. Contrary to previous research showing a relationship between emotion recognition and emotional contagion (e.g., [15]), participants in our study did not differ in emotional reactivity depending on emotion recognition. This suggests that the capacity to identify emotions based on their typical expression played a lesser role than emotion understanding and emotion management in the task used in this study. This may be explained by the instructions of the task, which emphasized perspective taking rather than emotion recognition. It is very likely that the participants, while watching the videos and listening to the protagonists’ stories, were more focused on understanding the general situation rather than attributing specific emotion to the narrators. On a related note, in the videos, the narrators were reporting past events therefore displaying subtle and mixed emotion expressions. This might have encouraged the participants to focus on other cues, typically the narrators’ discourse.

## Limitations

In the current study, we found indices of emotional hypersensitivity associated with high EI for positive narratives only. One possible explanation is that pleasant events generally do not need to be downregulated [49]. Conversely, emotional hypersensitivity in response to unpleasant narratives might be harder to detect because (implicit) regulatory processes might have operated simultaneously, inhibiting the activation of the muscle involved in frowning, and by extension, the subjective unfolding of emotional hypersensitivity. Further research is needed to test whether hypersensitivity to negative stimuli might be modulated by the type of emotion (e.g., sadness, fear, anger) and the type of appraisal (e.g., intensity and unexpectedness). More intense and unexpected emotional stimuli might increase the relevance of emotional stimuli– especially the negative ones– and promote stronger mimicry in people with high levels of emotional perception and understanding. Another way to reveal hypersensitivity to negative stimuli might be to increase the relationship between the expresser and the observer. Particularly, it has been shown that sadness expressions were preferentially mimicked when displayed by a close other [50]. Future research investigating this topic could measure emotional reactivity in a context closer to natural social interaction environment (e.g., interaction with a real person in real time or interaction with a friend rather than a stranger to share this kind of personal story).

On a methodological point of view, in the present study, facial muscles reactivity was operationalized as the average activation that occurred over a period of about 2 min. Over this period, a multitude of activations take place in response to various emotional stimuli. As a result, it was not possible to isolate the reactivity over time ― from the onset to the peak to the end of the recovery ― associated with each single emotional stimulus. To do this, it would be necessary to present a larger number of emotional stimuli of shorter duration (e.g., with an experimental design similar to the Dynamic Affective Reactivity Task [51]). A promising line of research would be to test whether individual differences in EI influence the reactivity over time. For example, people high on emotion perception and understanding might reach the peak faster and show a higher peak, while people high on emotion management might recover more quickly.

## Conclusion

The results of the current study shed some light about the hypersensitivity hypothesis according to which EI works by amplifying reactivity to emotional stimuli. First, they suggest that hypersensitivity effects might better be captured by implicit measures such as mimicry rather than explicit ones such as reporting of emotion. Second, there seems to be some influence of EI, specifically emotion understanding on facial muscles activity when watching positive videos and emotion management when watching negative videos. Although the results were not in line with our hypothesis, we think that they show some clear directions for further investigation of the hypersensitivity hypothesis.

Further research might address the relationship between hypersensitivity and regulation of emotion by employing different outcomes than those investigated here. As previously said, it is well possible that relatively automatic regulatory processing is deployed by hypersensitive people who are also emotionally intelligent. Evaluating consequences of such regulatory processes might allow us to better evaluate hypersensitivity and its implications. In addition, the question remains whether hypersensitivity is an asset in social behavior. For instance, if high EI individuals react more strongly to positive faces, does this way of functioning foster interpersonal relationships and ultimately increases their well-being? What would be the effect of stronger reactivity to negative faces? How can strong emotional reactivity to unpleasant emotions be disentangled from implicit emotion regulation? These open questions will help to fully understand under which conditions hypersensitivity is associated with EI, how hypersensitivity can be detected, and how it may ultimately impact important life outcomes.

## Electronic supplementary material

Below is the link to the electronic supplementary material.

Supplementary Material 1

## Data Availability

Data reported in this study and the scripts for statistical analyses can be found following the OSF link provided in the paper.

## Footnotes

1 The analyses of the m. zygomaticus major and m. corrugator supercilii were also run on the integral and led to the same effects.
