# Supplementary material for: How do emotionally intelligent individuals react to other people’s emotions? A study on emotional and facial reactions
Source: BMC Psychol. 2025 Mar 10;13:224. doi: 10.1186/s40359-025-02425-5 (PMC11895264; doi:10.1186/s40359-025-02425-5)
Supplement: Supplementary file 1 — Supplementary Material 1 [file 40359_2025_2425_MOESM1_ESM.pdf]

“How do emotionally intelligent individuals react to other people's emotions? A study on emotional and facial reactions”

Supplementary materials

Other measures collected in the study but not reported in the paper

As this study is part of a greater project on emotional intelligence and hypersensitivity, the online testing session contained several additional questionnaires that are not investigated in the present paper. Participants filled in the 24-item Brief HEXACO Inventory [1], a general question about their mood, the Highly Sensitive Person Scale [2, 3], the Perth Emotional Reactivity Scale short-form [4], the Interpersonal Reactivity Index [5, 6], the Cognitive Emotional Regulation Questionnaire short-form [7, 8], the Emotion Regulation Questionnaire [9, 10], the Compassion Scale [11], and the Subjective Happiness Scale [12, 13].

Procedure for the creation of the videos

We posted advertisements at a local university (in a different city from the one where testing would take place) and on social media, explaining the purpose of the videos (i.e., an emotional contagion task) and the fact that we were looking for women who would agree to talk about a positive and negative event they had experienced in their lives. Thirty-three women contacted us through an online questionnaire where they briefly reported two positive and two negative events they agreed to share on record. On this basis, we selected nine women whose emotional experiences were diverse and moderately intense. We then gave them more information about the goals of the study and what was needed from them during the recording. We asked them to prepare for the interview by thinking about the events, identifying their thoughts and feelings at that time, and being ready to talk about each event for approximately 1.5 to 2 minutes. The following week, the selected narrators came to the recording session which took place in a quiet room, one person at a time. After a short preparation time, they were seated on a stool before a

green background and the video recording started. The duration and the content of each emotional event were checked during the first recording and the narrators were asked to adapt their pace or the content when needed and when possible. Because the narrators had thoroughly thought about the events beforehand and because we wanted them to be as natural as possible, all videos were recorded in a maximum of two takes. Each narrator explained two negative and two positive emotional events (e.g., stress before an exam, a romantic break-up, a joyful vacation, succeeding in med school), allowing us to collect 36 videos. The videos were then edited: a grey background replaced the green one and videos were cropped to display only the narrator's face and shoulders.

In the second step, three of the authors selected a set of 12 videos (6 negative and 6 positive) based on their duration and their content. Videos with content that was judged as too intense (e.g., cancer diagnostic, physical assault) or not intense enough (e.g., likable work experience, annoying relationship) were eliminated. The pre-selected videos were then evaluated regarding their intensity and their valence by 40 French-speaking women aged between 19 and 34 (20 evaluators for the negative videos and 20 for the positive videos) on the online platform Prolific. The valence of each video was rated on an analog scale ranging from *0 = neutral* to *100 = completely positive/negative* (depending on the valence of the video) with a middle point at *50 = moderately positive/negative*. The intensity of the emotional experience was rated on an analog scale ranging from *0 = not intense at all* to *100 = extremely intense*. We also asked the evaluators to indicate the extent to which they were touched by the video, on an analog scale from *0 = not at all* to *100 = totally*. Based on these evaluations, six videos (3 positives and 3 negatives) were selected to be included in the emotional contagion experiment. The selected positive and negative videos had similar ratings of valence (M_{pos} = 89.6, sd_{pos} = 13.1, M_{neg} = 76.3, sd_{neg} = 20.9, $t(2.11) = -2.92$, ns.), intensity (M_{pos} = 80.4, sd_{pos} = 17.3, M_{neg} = 73.4, sd_{neg} = 22.1, $t(3.97) = -1.99$, ns.) and touching potential (M_{pos} = 69.8, sd_{pos} = 22.5, M_{neg} = 61.0, sd_{neg} = 27.6, $t(3.92) = -2.47$, ns.). Following a reviewer's suggestion, we evaluated the activation of the corrugator (AU04) and zygomaticus (AU12) muscles with OpenFace [14] and report it in Supplementary Table 1.

Supplementary Table 1

Characteristics of the videos used in the experiment.

Video	Valence	Content	Valence mean (SD)	Intensity mean (SD)	Touched mean (SD)	Video duration in seconds	Average presence of corrugator activity	Average presence of zygomaticus activity
1	N	Narrator explains her first year of med school, and how she was very stressed during her studies. She failed her first year twice, meaning that she was expelled from med school and had to give up on her dream.	69.25 (23.22)	70.80 (20.78)	56.10 (28.17)	113	0.74	0.03
2	N	Narrator was planning a nice weekend with her girlfriends when she discovered that her grandfather was lying in his bed, crying, and saying that he was going to die very soon. She explains her thoughts at that time.	75.10 (21.51)	70.90 (24.57)	61.70 (28.58)	106	0.74	0.00

3	N	Narrator explains that, following her anorexia diagnosis, she was placed in a psychiatric hospital without warning and against her will. She shares her feelings at that moment and while being in the hospital.	84.65 (14.95)	78.60 (20.83)	65.35 (26.59)	114	0.23	0.06
4	P	Narrator explains her first day of a sabbatical year in Australia, how she was feeling well when arriving there, and how everything was nice that day.	88.30 (11.05)	71.0 (22.53)	55.20 (26.60)	110	0.07	0.25
5	P	Narrator explains how, after failing one exam of her first year of med school, she eventually learned that she had passed. She develops her feelings of pride and happiness at that moment and how she will never forget it.	89.65 (10.82)	81.95 (13.08)	74.30 (19.03)	87	0.49	0.97
6	P	Narrator describes the bike she got when she was a teenager and how she enjoyed riding it. She explains how, one day in particular, she had a perfect moment of happiness.	90.95 (17.04)	83.45 (15.97)	68.55 (23.70)	125	0.03	0.53

Supplementary Table 2

Estimates from the models testing for the effect of the watching condition on subjective arousal (treatment contrast with ref = spontaneous condition).

	Subjective arousal		
Predictors	*Estimates*	*CI*	*p*
Intercept	4.60	4.28 – 4.92	**<0.001**
Condition - empathic vs. spontaneous	0.49	0.17 – 0.81	**0.003**
Condition - distancing vs. spontaneous	-0.16	-0.48 – 0.17	0.344
Observations	866		
Marginal R^2 / Conditional R^2	0.013 / 0.327		

Supplementary Table 3

Estimates from the models testing for the effect of the watching condition on subjective arousal (difference contrast).

	Subjective arousal		
Predictors	*Estimates*	*CI*	*p*
Intercept	4.71	4.46 – 4.97	**<0.001**
Condition - empathic vs. spontaneous	0.49	0.17 – 0.81	**0.003**
Condition - distancing vs. empathic	-0.65	-0.97 – -0.32	**<0.001**
Observations	866		
Marginal R^2 / Conditional R^2	0.013 / 0.327		

Supplementary Table 4

Estimates from the model testing for the effect of emotion understanding on subjective arousal in the spontaneous condition (H1).

	Subjective arousal		
Predictors	*Estimates*	*CI*	*p*
Intercept	4.60	4.31 – 4.88	**<0.001**
STEU (z-score)	-0.08	-0.37 – 0.21	0.579
Observations	288		
Marginal R^2 / Conditional R^2	0.001 / 0.109		

Supplementary Table 5

Estimates from the model testing for the effect of emotion recognition on subjective arousal in the spontaneous condition (H1).

	Subjective arousal		
Predictors	*Estimates*	*CI*	*p*
Intercept	4.58	4.29 – 4.87	**<0.001**
GERT (z-score)	0.07	-0.22 – 0.36	0.649
Observations	280		
Marginal R^2 / Conditional R^2	0.001 / 0.121		

Supplementary Table 6

Estimates from the model testing for the effect of emotion understanding on subjective arousal in the spontaneous and empathic conditions (H2).

	Subjective arousal		
Predictors	*Estimates*	*CI*	*p*
Intercept	4.85	4.58 – 5.11	**<0.001**
STEU (z-score)	-0.07	-0.33 – 0.20	0.624
Condition - empathic	0.50	0.18 – 0.83	**0.003**
STEU x Condition	0.01	-0.31 – 0.34	0.931
Observations	576		
Marginal R^2 / Conditional R^2	0.012 / 0.295		

Supplementary Table 7

Estimates from the model testing for the effect of emotion recognition on subjective arousal in the spontaneous and empathic conditions (H2).

	**Subjective arousal**		
Predictors	*Estimates*	*CI*	*p*
Intercept	4.84	4.57 – 5.11	**<0.001**
GERT (z-score)	0.01	-0.26 – 0.28	0.928
Condition - empathic	0.52	0.19 – 0.85	**0.002**
GERT x Condition	-0.11	-0.44 – 0.22	0.495
Observations	560		
Marginal R^2 / Conditional R^2	0.013 / 0.302		

Supplementary Table 8

Estimates from the model testing for the effect of emotion management on subjective arousal for in the spontaneous condition depending on the video valence (positive vs. negative) (H3).

	**Subjective arousal**		
Predictors	*Estimates*	*CI*	*p*
Intercept	4.61	4.32 – 4.90	**<0.001**
STEM (z-score)	0.04	-0.25 – 0.33	0.770
Valence - negative	0.33	-0.18 – 0.84	0.202
STEM x Valence	0.08	-0.43 – 0.59	0.754
Observations	284		
Marginal R^2 / Conditional R^2	0.006 / 0.130		

Supplementary Table 9

Estimates from the model testing for the effect of emotion management on subjective arousal for negative videos between the spontaneous and distancing conditions (H4).

	**Subjective arousal (negative videos)**		
Predictors	*Estimates*	*CI*	*p*
Intercept	4.68	4.34 – 5.01	**<0.001**
STEM (z-score)	0.13	-0.21 – 0.46	0.450
Condition - distancing	-0.26	-0.68 – 0.17	0.230
STEM x Condition	0.15	-0.28 – 0.57	0.499
Observations	285		
Marginal R^2 / Conditional R^2	0.007 / 0.434		

Supplementary Table 10

Estimates from the model testing for the effect of emotion management on subjective arousal for positive videos between the spontaneous and distancing conditions (H5).

	Subjective arousal (positive videos)		
Predictors	*Estimates*	*CI*	*p*
Intercept	4.41	4.06 – 4.76	**<0.001**
STEM (z-score)	-0.04	-0.39 – 0.30	0.803
Condition - distancing	-0.08	-0.48 – 0.33	0.713
STEM x Condition	-0.09	-0.49 – 0.31	0.667
Observations	285		
Marginal R^2 / Conditional R^2	0.001 / 0.502		

Supplementary Table 11

Estimates from the model testing for the effects of condition and valence on the zygomaticus activation (treatment contrast with ref set to spontaneous).

	**Inverse zygomaticus mean activation**		
Predictors	*Estimates*	*CI*	*p*
Intercept	-497.55	-529.54 – -465.55	**<0.001**
Condition - empathic vs. spontaneous	11.47	-12.25 – 35.19	0.343
Condition - distancing vs. spontaneous	-55.50	-79.30 – -31.71	**<0.001**
Valence - positive	169.42	135.84 – 203.00	**<0.001**
Condition (empathic vs. spontaneous) x Valence	64.05	16.61 – 111.50	**0.008**
Condition (distancing vs. spontaneous) x Valence	-37.19	-84.78 – 10.40	0.125
Observations	860		
Marginal R^2 / Conditional R^2	0.161 / 0.636		

Supplementary Table 12

Estimates from the model testing for the effects of condition and valence on the zygomaticus activation (difference contrast).

	**Inverse zygomaticus mean activation**		
Predictors	*Estimates*	*CI*	*p*
Intercept	-512.23	-541.19 – -483.26	**<0.001**
Condition - empathic vs. spontaneous	11.47	-12.25 – 35.19	0.343
Condition - distancing vs. empathic	-66.98	-90.75 – -43.21	**<0.001**
Valence - positive	178.38	158.97 – 197.78	**<0.001**
Condition (empathic vs. spontaneous) x Valence	64.05	16.61 – 111.50	**0.008**
Condition (distancing vs. empathic) x Valence	-101.24	-148.78 – -53.70	**<0.001**
Observations	860		
Marginal R^2 / Conditional R^2	0.161 / 0.636		

Supplementary Table 13

Estimates from the model testing for the effects of condition, valence and emotion understanding (STEU) on the zygomaticus activation in the spontaneous and empathic conditions.

	**Inverse zygomaticus mean activation**		
Predictors	*Estimates*	*CI*	*p*
Intercept	-491.72	-520.71 – -462.72	**<0.001**
STEU	-9.66	-38.69 – 19.37	0.512
Condition - empathic vs. spontaneous	11.26	-12.22 – 34.74	0.346
Valence - positive	201.26	177.78 – 224.74	**<0.001**
STEU x Condition (empathic. vs. spontaneous)	5.22	-18.28 – 28.72	0.663
STEU x Valence	23.22	-0.28 – 46.72	0.053
Condition (empathic vs. spontaneous) x Valence	64.50	17.54 – 111.46	**0.007**
STEU x Condition (empathic vs. spontaneous) x Valence	7.33	-39.67 – 54.33	0.759
Observations	575		
Marginal R^2 / Conditional R^2	0.187 / 0.640		

Supplementary Table 14

Estimates from the model testing for the effects of condition and valence on the corrugator activation (treatment contrast with ref set to spontaneous).

	Log corrugator mean activation		
Predictors	*Estimates*	*CI*	*p*
Intercept	-5.61	-5.67 – -5.54	**<0.001**
Condition - empathic vs. spontaneous	0.04	-0.00 – 0.08	0.069
Condition - distancing vs. spontaneous	0.02	-0.02 – 0.06	0.337
Valence - positive	-0.28	-0.34 – -0.22	**<0.001**
Condition (empathic vs. spontaneous) x Valence	-0.06	-0.14 – 0.02	0.169
Condition (distancing vs. spontaneous) x Valence	0.02	-0.06 – 0.10	0.645
Observations	863		
Marginal R^2 / Conditional R^2	0.095 / 0.715		

Supplementary Table 15

Estimates from the model testing for the effects of condition and valence on the corrugator activation (difference contrast).

	Log corrugator mean activation		
Predictors	*Estimates*	*CI*	*p*
Intercept	-5.59	-5.65 – -5.52	**<0.001**
Condition - empathic vs. spontaneous	0.04	-0.00 – 0.08	0.069
Condition - distancing vs. empathic	-0.02	-0.06 – 0.02	0.391
Valence - positive	-0.29	-0.32 – -0.26	**<0.001**
Condition (empathic vs. spontaneous) x Valence	-0.06	-0.14 – 0.02	0.169
Condition (distancing vs. empathic) x Valence	0.08	-0.01 – 0.16	0.067
Observations	863		
Marginal R^2 / Conditional R^2	0.095 / 0.715		

Supplementary Table 16

Estimates from the model testing for the effects of condition, valence and emotion management (STEM) on the corrugator activation in the spontaneous and distancing conditions.

	**Log corrugator mean activation**		
Predictors	*Estimates*	*CI*	*p*
Intercept	-5.59	-5.66 – -5.53	**<0.001**
STEM	-0.04	-0.10 – 0.03	0.284
Condition - distancing	0.02	-0.02 – 0.06	0.355
Valence - positive	-0.27	-0.31 – -0.23	**<0.001**
STEM x Condition	0.00	-0.04 – 0.04	0.937
STEM x Valence	0.07	0.02 – 0.11	**0.002**
Condition x Valence	0.02	-0.07 – 0.10	0.724
STEM x Condition x Valence	-0.04	-0.12 – 0.04	0.347
Observations	567		
Marginal R^2 / Conditional R^2	0.093 / 0.710		

References

1. de Vries RE. The 24-item Brief HEXACO Inventory (BHI). J Res Personal. 2013;47:871–80.

2. Aron EN, Aron A. Sensory-processing sensitivity and its relation to introversion and emotionality. J Pers Soc Psychol. 1997;73:345–68.

3. Bordarie J, Aguerre C, Bolteau L. Validation and study of psychometric properties of a French version of the Highly Sensitive Person Scale (HSPS-FR). Eur Rev Appl Psychol. 2022;72:100781.

4. Preece D, Becerra R, Campitelli G. Assessing Emotional Reactivity: Psychometric Properties of the Perth Emotional Reactivity Scale and the Development of a Short Form. J Pers Assess. 2019;101:589–97.

5. Davis MH. A multidimensional approach to individual differences in empathy. JSAS Cat Sel Doc Psychol. 1980;10:85.

6. Gilet A-L, Mella N, Studer J, Grühn D, Labouvie-Vief G. Assessing dispositional empathy in adults: A French validation of the Interpersonal Reactivity Index (IRI). Can J Behav Sci Can Sci Comport. 2013;45:42–8.

7. Garnefski N, Kraaij V. Cognitive Emotion Regulation Questionnaire – Development of a short 18-item version (CERQ-short). Personal Individ Differ. 2006;41:1045–53.

8. Jermann F, Van Der Linden M, d'Acremont M, Zermatten A. Cognitive Emotion Regulation Questionnaire (CERQ). Confirmatory factor analysis and psychometric properties of the French translation. Eur J Psychol Assess. 2006;22:126–31.

9. Gross JJ, John OP. Individual differences in two emotion regulation processes: Implications for affect, relationships, and well-being. J Pers Soc Psychol. 2003;85:348–62.

10. Christophe V, Antoine P, Leroy T, Delelis G. Évaluation de deux stratégies de régulation émotionnelle : La suppression expressive et la réévaluation cognitive. Eur Rev Appl Psychol. 2009;59:59–67.

11. Pommier E, Neff KD, Tóth-Király I. The development and validation of the compassion scale. Assessment. 2020;27:21–39.

12. Lyubomirsky S, Lepper HS. A measure of subjective happiness: Preliminary reliability and construct validation. Soc Indic Res. 1999;46:137–55.

13. Kotsou I, Leys C. Self-Compassion Scale (SCS): Psychometric properties of the French translation and its relations with psychological well-being, affect and depression. PloS One. 2016;11:e0152880.

14. Amos B, Ludwiczuk B, Satyanarayanan M. OpenFace: A general-purpose face recognition library with mobile applications. CMU School of Computer Science; 2016.
